# Polymer brush hypersurface photolithography

**DOI:** 10.1038/s41467-020-14990-x

**Published:** 2020-03-06

**Authors:** Carlos Carbonell, Daniel Valles, Alexa M. Wong, Andrea S. Carlini, Mollie A. Touve, Joanna Korpanty, Nathan C. Gianneschi, Adam B. Braunschweig

**Affiliations:** 1 Advanced Science Research Center at the Graduate Center of the City University of New York, 85 St Nicholas Terrace, New York, NY 10031 USA; 2 Department of Chemistry, Hunter College, 695 Park Ave, New York, NY 10065 USA; 3 PhD Program in Chemistry, Graduate Center of the City University of New York, 365 5th Ave, New York, NY 10016 USA; 4 Department of Chemistry, Northwestern University, Evanston, IL 60208 USA; 5 Department of Materials Science and Engineering, Northwestern University, Evanston, IL 60208 USA; 6 Department of Biomedical Engineering, Northwestern University, Evanston, IL 60208 USA; 7 PhD Program in Biochemistry, Graduate Center of the City University of New York, 365 5th Ave, New York, NY 10016 USA

**Keywords:** Polymer synthesis, Polymers, Synthesis and processing

## Abstract

Polymer brush patterns have a central role in established and emerging research disciplines, from microarrays and smart surfaces to tissue engineering. The properties of these patterned surfaces are dependent on monomer composition, polymer height, and brush distribution across the surface. No current lithographic method, however, is capable of adjusting each of these variables independently and with micrometer-scale resolution. Here we report a technique termed Polymer Brush Hypersurface Photolithography, which produces polymeric pixels by combining a digital micromirror device (DMD), an air-free reaction chamber, and microfluidics to independently control monomer composition and polymer height of each pixel. The printer capabilities are demonstrated by preparing patterns from combinatorial polymer and block copolymer brushes. Images from polymeric pixels are created using the light reflected from a DMD to photochemically initiate atom-transfer radical polymerization from initiators immobilized on Si/SiO_2_ wafers. Patterning is combined with high-throughput analysis of grafted-from polymerization kinetics, accelerating reaction discovery, and optimization of polymer coatings.

## Abstract (web summary)

Various lithographic approaches are being explored to create polymer brush patterns with micrometer-scale feature dimensions. Here the authors demonstrate a printing approach which allows independent control of the monomer composition and feature height of each pixel in a pattern, while circumventing the need for expensive photomasks.

## Introduction

Controlling the morphology and chemical composition of interfaces with micrometer-scale precision is a key challenge in surface chemistry, with repercussions from biology to optics and material science. Polymer brushes^1–3^, and particularly polymer brush patterns with micrometer-scale features, are increasingly explored in the context of microarrays^4–6^, smart surfaces^7–9^, and tissue engineering^10–13^ because their interfacial properties can be manipulated by tailoring the degree of polymerization, the monomer composition, and the grafting density of the polymer brushes. Of the many grafted-from polymerization reactions^14–16^, living polymerizations are ideal for patterning because the degree of polymerization is dependent upon reaction time, which affords precise control over the polymer height and block length^17–20^. Several challenges, however, have precluded the realization of the full potential of polymer brush patterning technologies. Typically, polymer brush coatings are composed of identical polymers uniformly distributed across the surface because no available printing tool is capable of controlling simultaneously polymer height and polymer composition at each position, while producing features with micrometer-scale dimensions. Also because the brushes on a surface are generally grown under identical conditions, a new surface must be prepared and characterized to determine the properties arising from each new set of reaction conditions^21–24^. This makes optimization of grafted-from reactions and developing structure–activity relationships time-consuming and costly. Therefore, there is a pressing need to develop printing platforms that (1) combine the feature resolution of modern lithography strategies with the ability to spatiotemporally localize a stimulus (e.g., light or heat) that induces the polymerization reaction, (2) can accommodate the demanding reaction conditions required to carry out advanced polymerization chemistries (e.g., in solution and O_2_ and H_2_O free), and (3) have the capability to independently vary the monomer composition of each pixel across the surface and along each polymer chain.

Various lithographic approaches are being explored currently in an effort to create polymer brush patterns with micrometer-scale feature dimensions and vary the composition of polymer brushes across the surface. For example, microcontact printing has been used to pattern self-assembled monolayers of initiators, and polymer brushes of uniform height and identical composition were grown upon immersing the substrate in monomer solution^25–27^. An alternative approach involves uniformly functionalizing the surface with the initiators and subsequently irradiating through a photomask, in the presence of the monomer solution to create a pattern^23^. This strategy can create gradients, which are important for tissue engineering^28,29^ or to study cell adhesion and migration^30–32^, but patterns containing multiple different polymers are printed with difficulty because photolithography generates one pattern per photomask, and serially aligning photomasks is tedious. Hawker et al. for instance have recently prepared patterns with four different polymer brush compositions following a multistep process, consisting of creating a polymer brush coating, modifying it through a photomask using click chemistry, and repeating this process several times to create patterns composed of up to four different polymer brush compositions^33^. Scanning probe lithographies^34,35^ have successfully produced polymer brush patterns by using the tip to deposit the initiator^36^, or creating a pattern with a resist, where, following resist development, the initiator is subsequently deposited^37^. Features as small as 25 nm are obtained, however, these methods uniformly irradiate the surface, so the resulting polymers are identical across the substrate. Electron-beam lithography has been successfully used for direct writing of polymer brushes with 20 nm feature dimensions^38^. Other strategies, such as self-assembly-driven growth of high-density polymer brush nanoarrays can reach feature sizes as small as 15 nm (ref. ^39^). Using massively parallel beam pen arrays, a monomer mixture was deposited onto a surface. Subsequently, irradiating the droplets produced glycopolymer arrays, where the polymer height in each position could be varied by manipulating the irradiation time, but each polymer was composed of the same monomer^40^. In an effort to create multiplexed polymer brush patterns using scanning probe technologies, we recently integrated microfluidics with beam pen lithography, but could only print a pattern composed of two different polymer compositions^41^. While each of these efforts is a milestone in polymer brush patterning, the goal of independently controlling height, composition, and feature dimensions for each pixel across the entire pattern, with micrometer resolution, and over a large (>1 mm^2^) area has remained elusive.

**Fig. 1:**
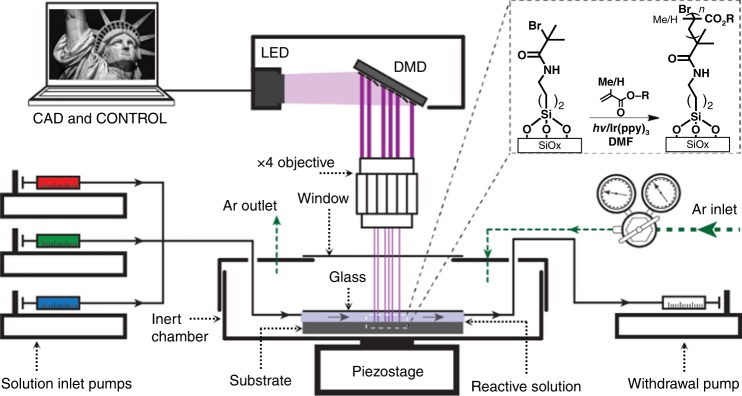
Photochemical printer. The printer uses microfluidics to introduce monomer solutions into the reaction chamber, a computer-controlled DMD to illuminate the surface, and a reaction cell mounted onto a piezoelectric stage to generate multicomponent polymer brush patterns by surface-initiated atom-transfer radical polymerization (inset).

Here, we report an approach toward printing combinatorial grafted-from polymer brush patterns, where the monomer composition and feature height of each pixel in a pattern can be controlled independently and with ~5 µm pixel edge length, while circumventing the need for expensive photomasks. We refer to these patterns as hypersurfaces—borrowing from its mathematical namesake to indicate a pattern where more than three properties of each pixel can be controlled independently (i.e*., x*- and *y-*position, height, and chemical composition along the chain). In addition, we use this terminology because four-dimensional printing has been adopted already to indicate additive manufacturing of objects whose shapes change over time in response to an external stimulus^42^. To create these hypersurfaces, we integrated a digital micromirror device (DMD), microfluidics, and an oxygen-free reaction chamber that was mounted onto a piezoelectric stage (Fig. 1). DMD-based printers have been combined already with microfluidics for oligonucleotide^43^ and oligopeptide^44^ microarray fabrication, and to prepare scaffolds for tissue engineering^45–47^. Our printer was built upon a TERA-Print E-series instrument, which coordinates the DMD (1024 × 768 independently controllable mirrors), light source (405 nm LED, 32 mW cm^−2^), and the piezoelectric stage with a CPU interface to project patterns taken from an uploaded image file onto a substrate. The inert atmosphere chamber is composed of a hermetically sealed polystyrene cell, with a glass window for passing light from the DMD to the surface, and inlet and outlet apertures for tubing that introduces the monomer solutions to the reactive substrate. An additional glass plate over the functionalized substrate forms a ~50 µL reaction cell, where the solutions are drawn over the surface by capillary forces^48^. Reactive solutions composed of monomers, solvent, and photosensitizer are introduced and withdrawn using syringe pumps that control flowrate within the reaction cell. A microfluidic chaotic mixer can be incorporated upstream to mix different proportions of components^48^.

## Results

### Optimization of surface-initiated ATRP printing

**Fig. 2:**
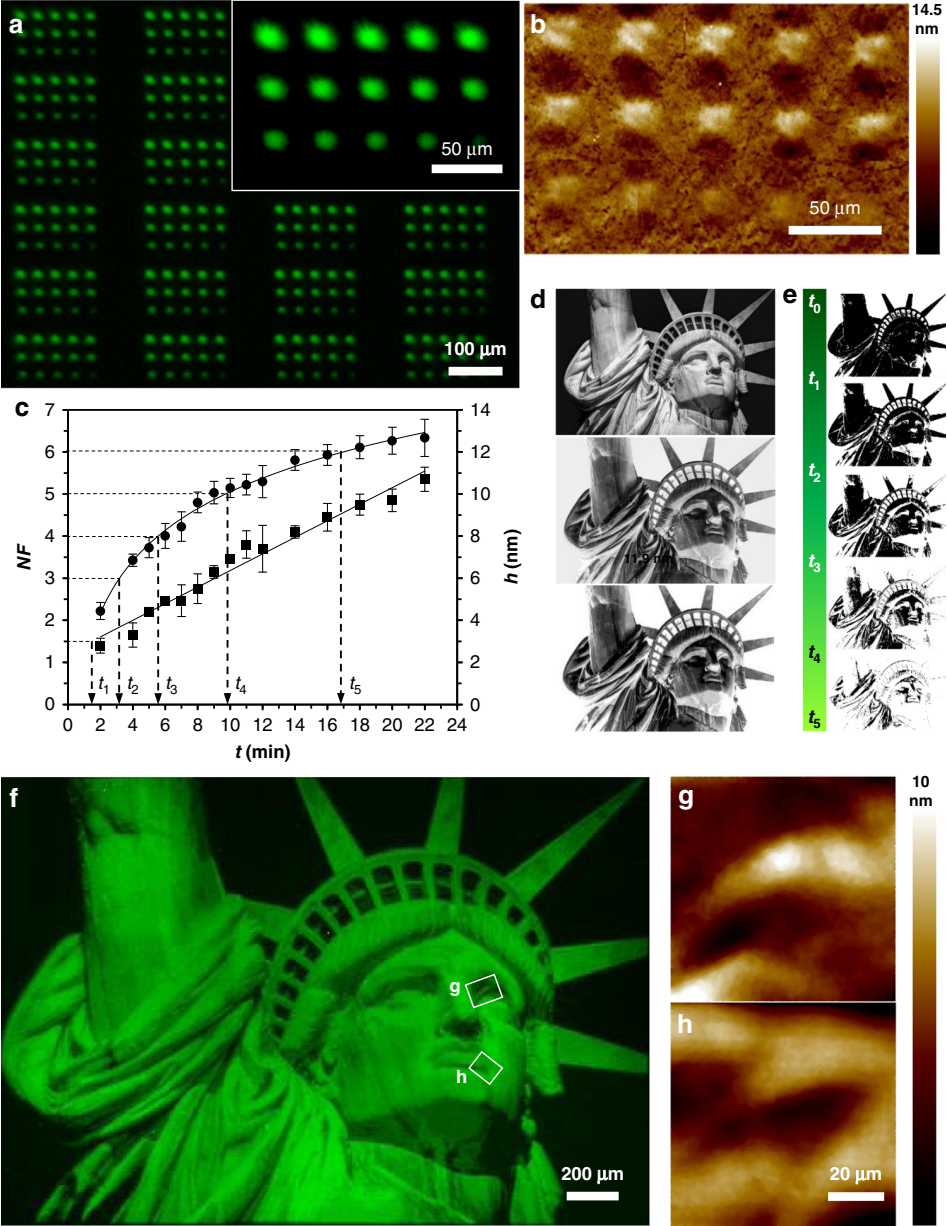
Control over height and position. **a** Fluorescence microscopy image (*λ*_ex_ = 451.5‒486.5 nm, *λ*_em_ = 540–580 nm) of 3 × 5 patterns consisting of polymer brush features printed at 15 different *t* to study the effect of *t* on *NF* and *h*. Inset is a magnified image of one of the arrays with *t* from 2‒22 min. **b** Composite of eight AFM height images corresponding to one of the arrays shown in **a**. **c** Dependence of *NF* (circles) and *h* (squares) with *t*. Error bars correspond to the standard deviation of five measurements for *NF* and three measurements for *h*. **d** Original black and white picture of Statue of Liberty (This image is not covered by the article CC BY license. Image credit to Oliver Kuehl. All rights reserved, used with permission.), inverted image, and converted to five gray levels, from top to bottom, respectively. **e** Threshold images uploaded to the CPU controller corresponding to the five different *t* required to obtain a polymer brush pattern (300:1 MMA:FMA) with five different *NF* and *h* levels shown in **f**. **g**, **h** AFM height measurements from the areas marked with white boxes in **f**.

We first sought to demonstrate the ability to generate polymer brush patterns with independent control over height at each pixel using photoinduced surface-initiated atom-transfer radical polymerization (SI-ATRP). This reaction was selected for these proof-of-concept studies because of its broad monomer scope and narrow molecular weight distribution^49^, and, because of these advantageous attributes, it is increasingly used to generate grafted-from polymer coatings and patterns. Typically, to study the kinetics of a grafted-from polymerization, each data point requires printing and analyzing a separate surface, which is time-consuming and subject to substantial experiment-to-experiment variation^23,50^. Here, we study multiple different polymerization conditions in a single print to rapidly determine the relationship between irradiation time (*t*) and polymer height (*h*). First, the reactive substrate was prepared by immersing a freshly cleaned Si/SiO_x_ wafer in an aminopropyltriethoxysilane solution to generate amine-functionalized wafers that were subsequently reacted with α-bromoisobutyryl bromide overnight to create a surface uniformly coated with the SI-ATRP initiator (Supplementary Fig. 1). Each step in this process was characterized by X-ray photoelectron spectroscopy (XPS) and contact angle measurements, and the data were consistent with the proposed surface reactions (Supplementary Fig. 2). The substrate was introduced into the inert chamber inside a glovebox, where the solutions are prepared with distilled dimethylformamide (DMF) and degassed monomers. Approximately 50 µL of a DMF solution containing methyl methacrylate (MMA), fluorescein *O*-methacrylate (FMA), and Ir(ppy)_3_ was deposited onto the surface and a cover glass was placed over the droplet. The chamber was then sealed, removed from the glovebox, and mounted onto the piezostage of the printer. FMA was added to MMA in a molar ratio 1:300 so that the patterns could be analyzed via fluorescence microscopy. A pattern of 15 features, irradiated at times ranging from 2–22 min, repeated 121 times across the 4.4 × 3.3 mm printing area, was sequentially projected onto the surface with the DMD. Following printing, the surfaces were washed with EtOH and sonicated for 5 min in DMF to remove any polymer that was not grafted to the surface, and the resulting polymer brush patterns were analyzed by fluorescence microscopy and atomic force microscopy (AFM; Fig. 2). Both *h* and normalized florescence (*NF* = feature fluorescence/background fluorescence) increase with increasing *t*. The feature *h* varied from 2.8 ± 0.4 nm to 10.7 ± 0.6 nm for 2 min and 22 min, respectively. The *NF* ranges from 2.2 ± 0.2 to 6.3 ± 0.4. The linear evolution of the polymer brush height with time is consistent with the kinetics measured in other studies of surface-initiated ATRP polymerizations^23,51^. Interestingly *NF* shows a logarithmic evolution, which may be the result of increasing adsorption of emitted light as the polymers grow (Fig. 2c). The printing was repeated under continuous flow at 5 µL/min to determine how flow affected printing, using the microfluidics to control the flowrate across the substrate, and these prints behave in a similar way (Supplementary Fig. 3).

To confirm whether these features were the result of poly(methyl methacrylate) (PMMA) polymerization, substrates prepared under the same conditions were analyzed with XPS (Supplementary Fig. 4). In addition, both unbound and surface-bound polymers were characterized by matrix-assisted laser desorption ionization imaging mass spectrometry (MALDI-IMS). First, unbound polymer was generated in the photochemical printer with irradiation times of 1, 2, 5, 10, and 40 min, then analyzed by MALDI-IMS to determine mass distributions and peak spacings (Supplementary Fig. 5a). A non-Gaussian distribution of peaks with spacings of 100 Da was observed, corresponding to the MMA monomer mass. Unfortunately, the non-Gaussian distribution, most likely a result of fragmentation of PMMA by MALDI-MS (ref. ^52^), prevented the quantification of polymer molecular weight distribution and dispersity. However, overall increases in signal intensity were observed for polymers generated under longer irradiation time (e.g., 10 vs. 40 min) up to the measured range of 20,000 Da, suggesting that higher molecular weight polymers were generated with longer irradiation and fragmenting during MALDI analysis. Next, patterned substrates with polymer covalently bound to the surface were analyzed (Supplementary Fig. 4b). Similar to the studies with the unbound PMMA polymer, uniform peak spacings were observed with a non-Gaussian distribution. We note that the peak spacings observed for the patterned substrate were 107 Da. It is currently unclear as to why the spacings of the polymer units are increased, but because the spectral peaks themselves are also broader, we believe these observations may be a result of a difference in ionization of the covalently bound polymer by the MALDI instrument, in comparison to the unbound polymer, despite identical sample preparation and ionizing conditions. Definitive polymer signal was detected at irradiation times as low as 1 min, with increasing mass signal intensity over time. This is consistent with changes in height measured by AFM and fluorescence signal intensity changes over time (Fig. 2c). Overall, MALDI-IMS definitively identified polymer generated within our photochemical printer, whether the polymer was unbound or covalently bound to a substrate surface.

A major challenge in polymer brush lithography is creating gradients, where brush density or brush height is varied across the surface. Here, we show how the kinetic data described above are used to create such a gradient pattern, where the *h* at each pixel was controlled independently to print an image of the Statue of Liberty. To do so, we chose a black and white picture of the Statue of Liberty (Fig. 2a) that was converted to a 1074 × 768 bitmap image, so each DMD mirror projects a single pixel with a corresponding width of 4.3 μm. The image was then inverted (Fig. 2b) and converted to five levels of gray (Fig. 2c), and finally, five black and white images based upon five threshold levels (Fig. 2d) were created. Each black pixel of each image is translated by the instrument into one mirror projecting light to the surface. To create the images, we start with all the black pixels ON at *t*_0_ = 0 (Fig. 2e), and we sequentially turn mirrors OFF to obtain polymers of different heights at the desired positions. Finally, all mirrors are turned OFF at *t*_5_. The last mirrors turning OFF, in *t*_5_, are the ones that have been ON during all the process and for a total time of *t*_5_, which corresponds to the longest exposure time (i.e., longest polymers). Using the kinetic fit obtained in Fig. 2c, we calculated the exposure times (*t*_0_ = 0; *t*_1_ = 1.37; *t*_2_ = 3.17; *t*_3_ = 5.55; *t*_4_ = 9.70; and *t*_5_ = 16.97 min) required at each pixel for obtaining the selected *NF* values (1.5, 3, 4, 5, and 6). We then sequentially printed the series of five images to produce a pattern, where the last image corresponds to the higher/brighter layer. Figure 2f shows a composite of nine fluorescence microscopy images of the polymer brush pattern obtained after rinsing the substrate with EtOH and sonicating in DMF for 10 min. AFM measurements of the marked areas in Fig. 2f (Fig. 2g, h) confirm that the different *NF* intensities correspond to polymer brushes of different *h*. The smallest isolated pixel size obtained, however, is 10 μm as a result of light dispersion, although with larger feature dimensions, light dispersion is not an issue as 100 mirrors project a pattern of 430 μm.

### Grafted-from polymer brush hypersurfaces

Another major challenge we overcome with this platform is the ability to simultaneously control both *h* and monomer composition at each pixel. That is, create hypersurfaces with potentially thousands of different polymer brushes in the array. Such patterns could be important for applications ranging from microarrays to modeling and understanding interactions of polymers with cells, bacteria, and viruses.

**Fig. 3:**
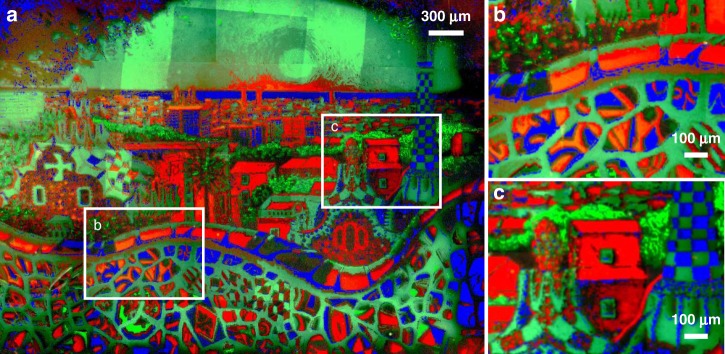
Multicomponent polymer brush patterns. **a** Composite of 75 microscopy images (25 images per channel) showing all three different fluorophores utilized to print an image of the Barcelona skyline (courtesy of Ana Maria Edulescu). **b** and **c** magnification of the areas marked in **a**.

We produced a multicomponent pattern by sequentially introducing three differently colored fluorescent monomer solutions into the reaction cell. This required first calibrating printing conditions for copolymers containing red (methacryloxyethyl thiocarbamoyl rhodamine B (RMA)) and blue ((trifluoromethyl)coumarin]methacrylamide (CMA)). First, the relationship between *NF* and *t* was studied for CMA and RMA mixtures at 223:1 and 5000:1 molar ratios to MMA, respectively, following the same protocol as described above. The *NF*_max_ values were 6.5 ± 0.6 for RMA and 1.12 ± 0.02 for CMA (Supplementary Fig. 6). The low *NF* observed on the CMA-based brushes is likely because of the lower incorporation of CMA into the PMMA brush because of the slower polymerization of acrylates compared to methacrylates in free radical polymerizations^53^. To create the multicolor pattern, a painting of Barcelona was decomposed into three channels (red, green, and blue, Supplementary Fig. 7), each of which was subsequently assigned to a monomer (RMA:MMA, FMA:MMA, and CMA:MMA mixtures). These three images were then processed following the same procedure described for the printing of the Statue of Liberty, but with four threshold levels per channel (Supplementary Fig. 8). Three DMF solutions containing FMA, RMA, and CMA were prepared and sequentially flowed at 5 µL/min over the substrate, while each solution was exposed to the corresponding pattern, followed by 5 min of rinsing at 100 µL/min upon changing solutions. Figure 3 shows a composite of 75 fluorescent microscopy images of the pattern (25 images per channel, see Supplementary Fig. 9). The processing of the merged images is explained in Supplementary Methods and Supplementary Fig. 10. This print demonstrates that, in principle, an unlimited number of monomer compositions and mixtures can be sequentially introduced onto the printing platform to control the polymer composition and *h* at each pixel, thereby overcoming a major limitation in polymer brush lithography.

### Block copolymer hypersurfaces

**Fig. 4:**
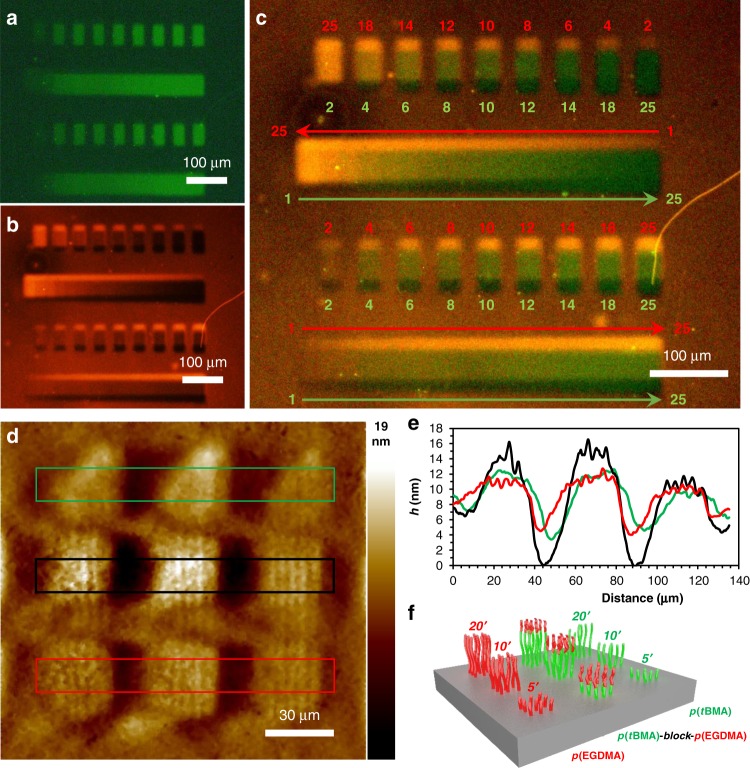
Block copolymer arrays. **a** Fluorescence microscopy image (*λ*_ex_ = 530–550 nm, barrier filter *λ*_em_ = 575 nm) of a *p*(*t*BMA-FMA) (300:1) pattern composed of four rows printed at 1–25 minutes (left to right). **b** Fluorescence microscopy image (*λ*_ex_ = 530–550 nm, barrier filter *λ*_em_ = 575 nm) of the same area as **a** showing a *p*(EGDMA-RMA) (6000:1) pattern composed of four rows printed at 1–25 min from right to left (top rows) and left to right (bottom rows). **c** Composite of **a** and **b**. **d** AFM height image of an array composed of *p*(*t*BMA) brush features (triangles and middle row) printed at 5, 10, and 20 min *t* from right to left. Bottom row is composed of *p*(EGMA) brushes which were also printed over the *p*(*t*BMA) on middle row at 5, 10, and 20 min from right to left. **e** Average height profiles of bands marked by colored squares in **d**.

Multiplexed block copolymer patterns were printed, where the monomer composition at each *x*- and *y*-coordinate, as well as along the chain, was independently controlled, a challenge for which there exists no solution amongst micro- and nanolithography methods. In this pattern, each pixel contained brushes composed of either poly(ethylene glycol dimethacrylate) (*p*EGDMA), poly(*tert-*butyl methacrylate) (p*t*BMA), or block copolymers of the two. The blocks are printed by sequential introduction of the monomer solutions into the reaction cell, and the *h* of each block is a function of the *t*. In the first of these patterns, EGDMA and *t*BMA doped with FMA (300:1) and RMA (6000:1), respectively, were printed into homopolymer and block copolymer brushes that could be analyzed by fluorescence microscopy. The pattern consisted of four rows, in which first and third rows (from top to bottom) were printed forming nine features at nine different *t* (2–25 min). The second and fourth rows were printed to form a continuous feature at 18 different *t* (2–25 min) (Fig. 4a). Then, *p*(*t*BMA-*co*-RMA) was printed partially overlapped with the *p*(EGDMA-*co*-FMA) pattern (Fig. 4b). The composite fluorescence microscopy image (Fig. 4c) shows 18 different proportions of *p*(EGDMA-*co*-FMA)-*block*-*p*(*t*BMA-*co*-RMA) in rows 1 and 3 and two different gradients in rows 2 and 4. The colocalization of the two dyes is evidence of block copolymer formation.

Another pattern was printed for analysis by AFM, where the changes in *h* confirmed the formation of block copolymer brushes. To this end, 432 repeats of a 3 × 3 pattern composed of rows of *p*(*t*BMA), *p*(EGDMA), and *p*(*t*BMA)-*block*-*p*(EGDMA) were printed (Fig. 4). First, in the top and middle rows of this pattern, features of *p*(*t*BMA) were grown with *t* of 5, 10, and 20 min. Then, the reaction cell was rinsed with an EGDMA solution in DMF at 100 µL/min for 2 min. The EGDMA was then patterned at same *t* in the middle and bottom rows, where in the middle row the *p*(*t*BMA) blocks grow from the living *p*(EGMA) block. Figure 4d shows AFM analysis of one of these 3 × 3 patterns. In the top and bottom row, which are the *p*(*t*BMA) and *p*(EGDMA) homopolymers, respectively, *h* increases with increasing *t*. The middle row, where the blocks were grown on top of each other, *h* is greater than either the top or bottom row, indicating that the chain ends remain living, and that the polymers continue to grow upon the introduction of the *t*BMA.

## Discussion

We have developed a platform for the photochemical patterning of block copolymer arrays, where the position and composition of each of the > 750,000 pixels can be independently controlled, and with micrometer-scale feature resolution. Because the surface is irradiated by a computer-modulated DMD, arbitrary patterns can be printed without necessitating an expensive series of photomasks. The integration of microfluidics and an air-free reaction chamber with the DMD is the key innovation that allows the spatiotemporally controlled grafting of different materials onto the substrate, and could, in principle, be used to make polymer patterns composed of a practically unlimited number of unique brush compositions. Although living SI-ATRP offers broad monomer scope and narrow molecular weight distribution, it is also challenging since it requires oxygen-free conditions, but the environmental control enabled by the air-free reaction cell means that this platform is compatible with even the most demanding photochemical reactions. We also demonstrate the ability to quantify polymer brush kinetics in a single print, and have printed random- and block copolymer microarrays, where in the latter, monomer composition along the chain was carefully regulated. While the SI-ATRP polymerization was studied here, this printer is a general tool for combinatorial surface photochemistry, and the facility with which hundreds or even thousands of different reactions conditions can be attempted in each print promises to rapidly accelerate progress in research disciplines where interfacial organic composition has a critical role. In the future, we will explore different chemistries, automate the microfluidics to coordinate them with the DMD, and integrate beam pen arrays^54^ to improve throughput, resolution, and versatility. Ultimately, we envision a new era of soft lithography where the fabrication of synthetic surfaces with complexity comparable to what is found in biological interfaces will soon become a reality.

## Methods

### Substrate functionalization

All materials were purchased from VWR or Fisher unless otherwise noted. Four-inch <100> silicon wafers with 500 nm thermal oxide layer (Nova Electronic Materials, USA) were cleaned in piranha solution (3:1 H_2_SO_4_:H_2_O_2_) for 15 min, and rinsed with Milli-Q water (18 MΩ). The wafer pieces were then functionalized with (3-aminopropyl)triethoxysilane (Gelest, USA) following a previously reported method^55^. These amine-terminated substrates were immersed in 120 mL of CH_2_Cl_2_ with 1.67 mL (12 mmol) Et_3_N for 30 min. A total of 1.48 mL (12 mmol) of α-bromoisobutyryl bromide was then added, and the solutions were kept for 18 h in the dark. Substrates were then rinsed with CH_2_Cl_2_, EtOH, and Milli-Q H_2_O, dried with an air gun, and stored in an Ar atmosphere in the dark until used.

### Printing solutions

FMA (Millipore-Sigma, USA) was purified through a silica column (SiO_2_ CH_2_Cl_2_:MeOH 97:3). Methacryloxyethyl thiocarbamoyl rhodamine B (RMA) (Polysciences Inc., USA) was purified through a silica column (SiO_2_ CH_2_Cl_2_:MeOH 98.5:1.5). Inhibitor was removed from MMA, EGDMA, and *t*BMA (Alfa-Aesar, USA) by flowing each monomer through a short plug of basic alumina. After the alumina plug, monomers were degassed in a vial wrapped with aluminum by bubbling Ar for 1 hour. 7-[4-(Trifluoromethyl)coumarin]methacrylamide (CMA) (Millipore-Sigma, USA), and Tris[2-phenylpyridinato-C^2^,*N*]iridium(III) (Ir(ppy)_3_) (Millipore-Sigma, USA) were used without further purification. Printing solutions were prepared as follows: Ir(ppy)_3_ (1.82 mM in distilled DMF) stock solution was first prepared and kept in the glovebox. FMA solution (24.9 mM FMA, 7.5 M of MMA, and 0.36 mM of Ir(ppy)_3_ in DMF), RMA (1.25 mM RMA, 7.5 M MMA, and 0.61 mM of Ir(ppy)_3_ in DMF), CMA (33.6 mM CMA, 7.5 M MMA, and 0.36 mM Ir(ppy)_3_ in DMF), EGDMA (4.24 M EGDMA, 0.364 mM of Ir(ppy)_3_ in DMF), and *t*BMA (4.92 M *t*BMA and 0.364 mM of Ir(ppy)_3_ in DMF) stock solutions were prepared for printing.

### Photochemical patterning

The printer uses a TERA-Fab E Series instrument (TERA-print, USA) equipped with DMD containing 1024 × 768 micromirrors, a 405 nm LED light source (32 mW cm^−2^), and a piezoactuated *x*, *y*, and *z* platform. An air-free chamber was made with two polystyrene dishes, equipped with an Ar inlet with a valve, and two apertures for the microfluidic tubing connection. After setting the reactive substrate inside the glovebox, the valve is closed and the chamber is sealed with Parafilm, including inlet/outlet apertures for the microfluidics. Once out of the glovebox, the Ar inlet is plugged to a T connection with two more valves for purging of the air from the tubing. After purging the gas lines, the Ar valve is opened, the tubing seals are removed, and the PEEK 1/16˝ tubing introduced. The solutions are then flowed into the reactive droplet using NE-1000 Programmable Syringe Pumps (Pump Systems Inc., USA). All prints were performed at 8 mW cm^−2^. The *t* used to print Fig. 2a were: 2, 4, 5, 6, 7, 8, 9, 10, 11, 12, 14, 16, 18, 20, and 22 min.

## Supplementary information

Supplementary Information

## Data Availability

The data that support the findings of this study are available from the corresponding author upon reasonable request.
