# Supplementary material for: Polymer brush hypersurface photolithography
Source: Nat Commun. 2020 Mar 6;11:1244. doi: 10.1038/s41467-020-14990-x (PMC7060193; doi:10.1038/s41467-020-14990-x)
Supplement: Supplementary file 1 — Supplementary Information [file 41467_2020_14990_MOESM1_ESM.pdf]

Supplementary Information

Polymer Brush Hypersurface Photolithography

Carbonell, C. *et al.*

Table of contents:

1. **Substrate functionalization** **3**
2. **Printing under continuous flow** **5**
3. **XPS C *1s* analysis** **6**
4. **MALDI-MS Analysis** **7**
5. **RMA and CMA printing optimization** **9**
6. **Multicolor printing** **10**
7. **Supplemental Methods: MALDI-MS & image treatment** **12**
8. **Calculation of grafting density** **15**
9. **References** **16**

1. Substrate functionalization

All materials were purchased from VWR or Fisher unless specially noted. 4-inch <100> silicon wafers with 500 nm thermal oxide layer (Nova Electronic Materials, USA) were cleaned in piranha solution (3:1 H_2SO_4:H_2O_2) for 15 min, and rinsed with Milli-Q water (18 MΩ). The wafer pieces were then functionalized with (3-aminopropyl)triethoxysilane (APTES) (Gelest, USA) following a previously reported method. These amine-terminated substrates were immersed in 120 mL of CH_2Cl_2 with 1.67 mL (12 mmol) triethylamine for 30 min. 1.48 mL (12 mmol) of 2-bromoisobutyryl bromide were then added, and the solutions were kept for 18 h in the dark. Substrates were then rinsed with CH_2Cl_2, EtOH, and Milli-Q H_2O, dried with an air gun, and stored in an Ar atmosphere in the dark until used.

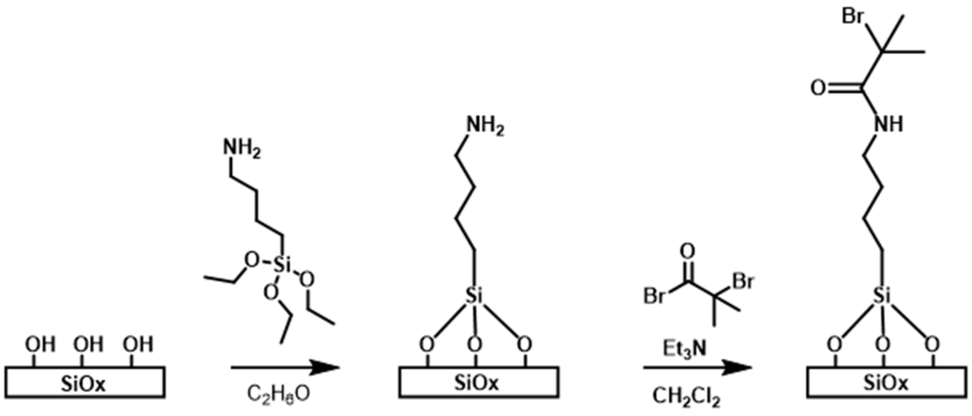

Supplementary Figure 1. Surface functionalization

Characterization of functionalized surfaces. Contact angles were taken with a home-made platform. A droplet of water was deposited onto the surface with a syringe. An image of the water droplet on the surface was taken with a USB microscope camera (MixMart, UK). Contact angles were evaluated using the “Contact Angle” plugin in ImageJ.[1] Errors are reported as standard deviation of five measurements taken from five different batches. XPS data were taken using Physical Electronics VersaProbe II XPS.

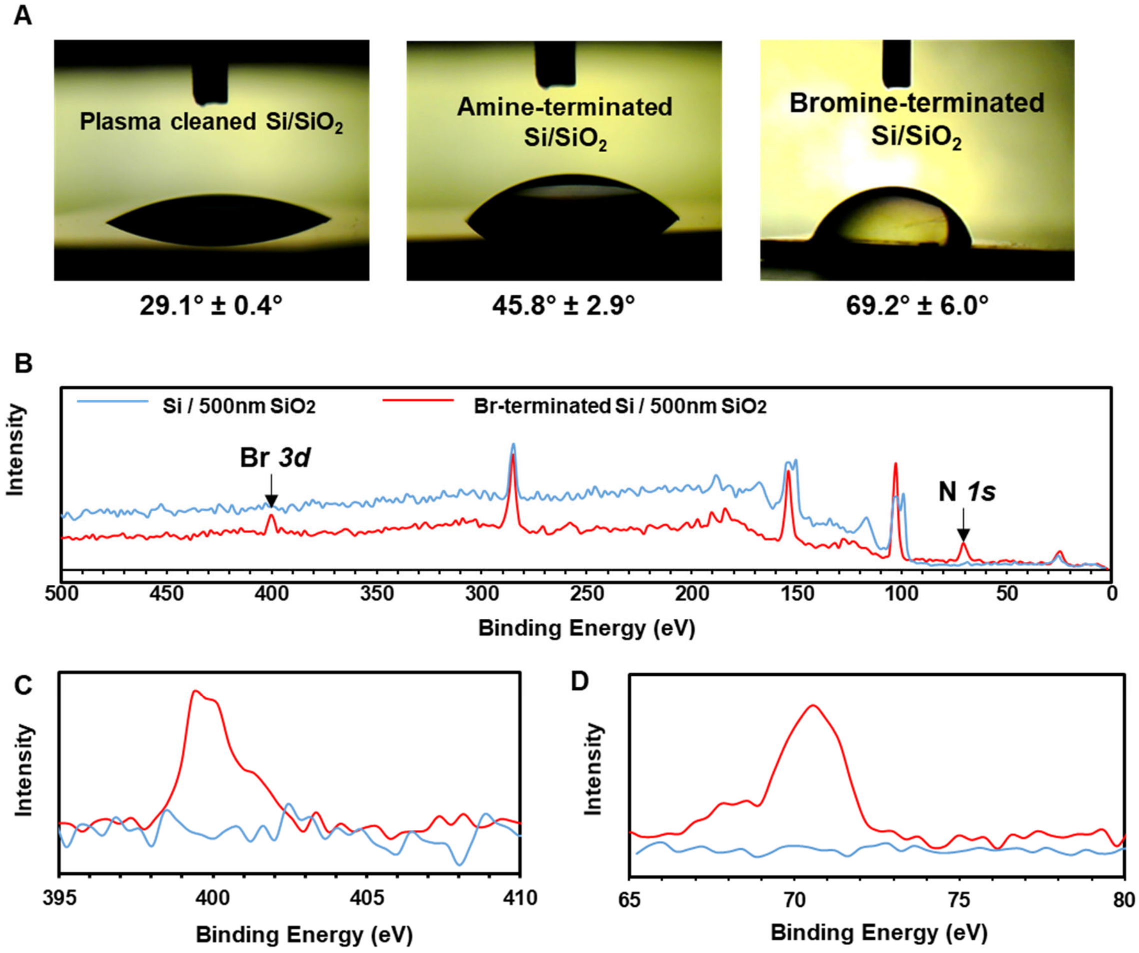

Supplementary Figure 2. Contact angle and XPS measurements of functionalized substrates. (**A**) Contact angles of plasma cleaned Si/SiO_2, amine-terminated Si/SiO_2 and bromine-terminated Si/SiO_2. (**B**) X-ray photoelectron spectra of surfaces before and after functionalization. (**C**) Br (Br *3d*) peak indicates successful functionalization with α-bromoisobutyryl bromide. (**D**) Nitrogen (N *1s*) peak indicates successful functionalization with APTES.

2. Printing under continuous flow

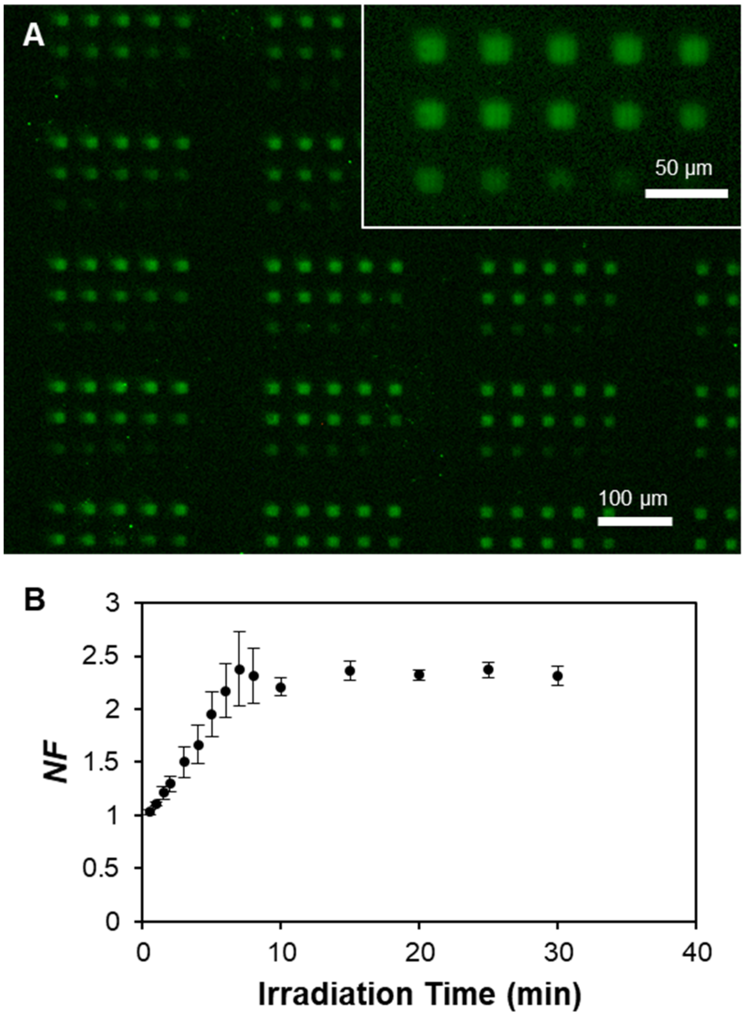

Supplementary Figure 3. Kinetic study of printing under continuous flow. A pattern of 15 features of FMA to MMA in a molar ratio 1:300, irradiated at times ranging from 2 – 22 min and repeated 121 times across the 4.4 x 3.3 mm printing area under continuous flow (5 µL/min). (**A**) Fluorescence microscopy image (λ_{ex} = 451.5 – 486.5 nm, λ_{em} = 540 – 580 nm) of 3 x 5 patterns consisting of polymer brush features printed at 15 different illumination times to study the effect of *t* on *NF.* Inset is a magnified image of one of the arrays. (**B**) Dependence of *NF* and *h* with time. Error bars correspond to the standard deviation of five measurements for *NF*.

3. XPS Analysis

C *1s* XPS data was taken with a Physical Electronics VersaProbe II XPS using an Al monochromatic X-ray source (1486.6 eV) at 37.6 W and Neutralizer gun operating at 2.0 V and 20 μA. Beam diameter was set to 200 μm, time per data point to 10 s at intervals of 0.125 eV, and pass energy at 29.35 eV.

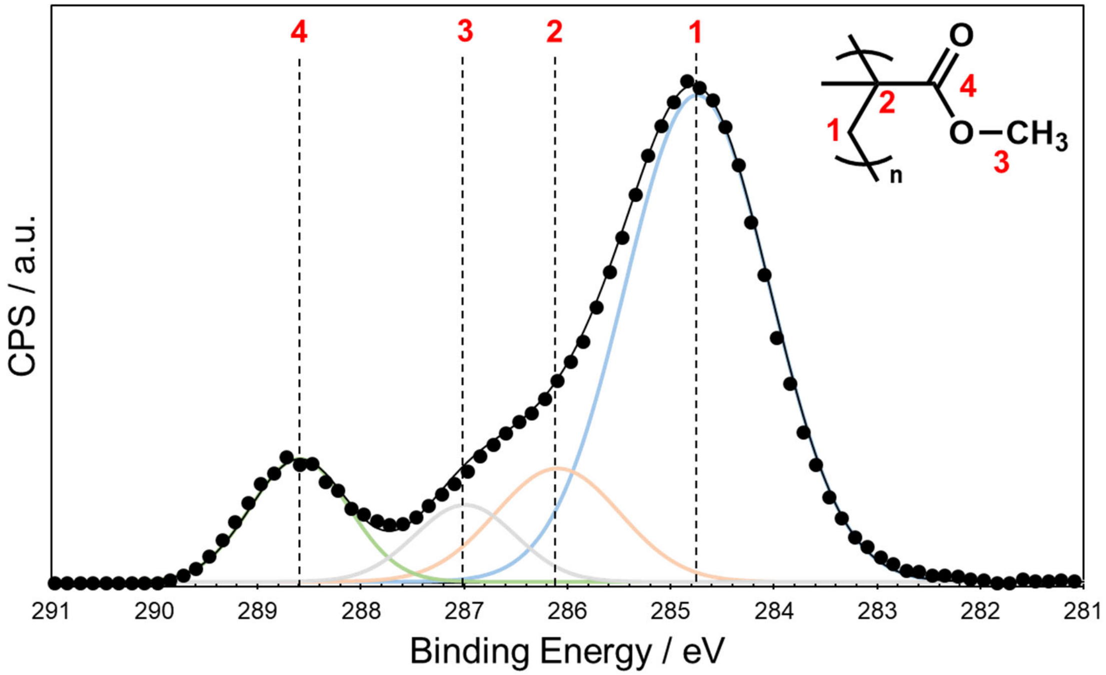

Supplementary Figure 4. XPS analysis of polymer-coated surface. C *1s* XPS spectrum (dots) of PMMA brushes showing the characteristic peaks of PMMA, correlated with the specific C bonds in red numbers.

4. MALDI-MS Analysis

Matrix-assisted laser desorption ionization imaging mass spectrometry (MALDI-IMS) was utilized to analyze mass distributions on poly(methyl methacrylate) (PMMA) photopolymerized surfaces (**Supplementary Figure 5**). A laser focus diameter of 50 µm was raster-scanned across each surface to produce a mass spectrum at each dwell location on the surface, then each spectrum was compiled to generate a 2D map of mass (m/z) signals. Solutions of PMMA were irradiated for varying lengths of time (1 - 40 min), spotted and dried onto a MALDI substrate, and evenly coated with matrix. By MALDI-IMS analysis, polymer signal was not detected below 5 mins of irradiation, presumably because of the low polymer concentrations within the bulk reaction solution. However, for longer irradiation times, polymer was detected, with uniform peak spacings of 100 Da attributable to that of methyl methacrylate units. The lack of a Gaussian distribution of masses within the analyzed mass range of 50-20,000 Da is consistent with literature reports of fragmentation patterns with PMMA by MALDI MS analysis. Furthermore, longer irradiation times resulted in an overall increase in signal intensity across the analyzed mass range. In particular, signal intensity increases within the 4000 - 8000 m/z regime were easily observed. Colorized 2D maps using a mass filter of 5000 (± 500) m/z were generated to highlight signal intensity changes with irradiation time (**Supplementary Figure 5 a, b**).

The next step was to photoprint a pattern of six square regions of PMMA covalently bound to a substrate surface, where each region was irradiated for a different length of time to recapitulate the varying polymerization conditions used to generate the patterns in **Figure 2**. Polymer signal containing similar fragmentation patterns to that of the unbound polymer samples (see above) were observed.

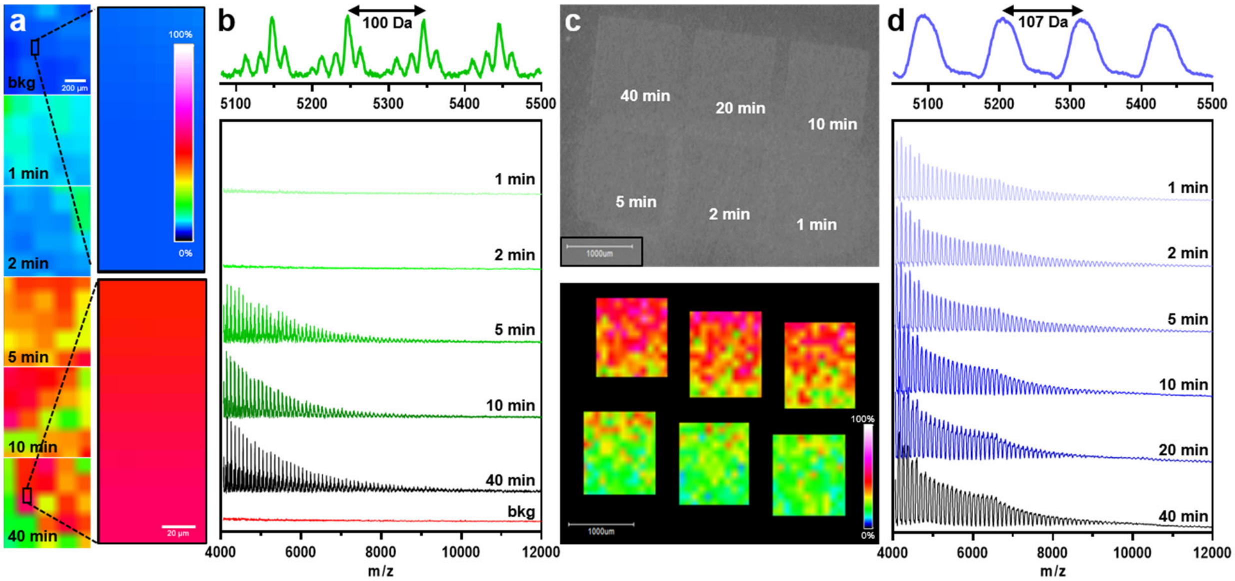

Supplementary Figure 5. MALDI-IMS of PMMA constructs. (a) MALDI-IMS map of unbound polymer generated in the photochemical printer after 1, 2, 5, 10, and 40 min irradiation. Colorized maps based on integrated mass signal within the range of 5000 (± 500) *m/z*. Insets are magnified arrays of matrix background (top) or 40 min irradiated polymer (bottom). Scale bars are 200 µm and 20 µm, respectively. **(b)** Mass spectral peak spacings of 100 Da corresponds to methacrylate units within PMMA (top). Averaged MALDI spectra of corresponding maps (bottom). **(c)** Optical

image (top) of surface-bound polymer generated by the photochemical printer after 1, 2, 5, 10, 20, and 40 min irradiation. Corresponding colorized maps (bottom) based on mass filtering for 5000 (± 500) *m/z*. Scale bar 1000 μm. **(d)** Mass spectral peak spacings of 107 Da correspond to monomer units of printed polymer (top). Averaged MALDI spectra of corresponding maps (bottom). All MALDI-IMS maps were generated as 2D plots of 50 μm diameter pixels, each of which corresponds to a single mass spectrum.

5. RMA and CMA printing optimization

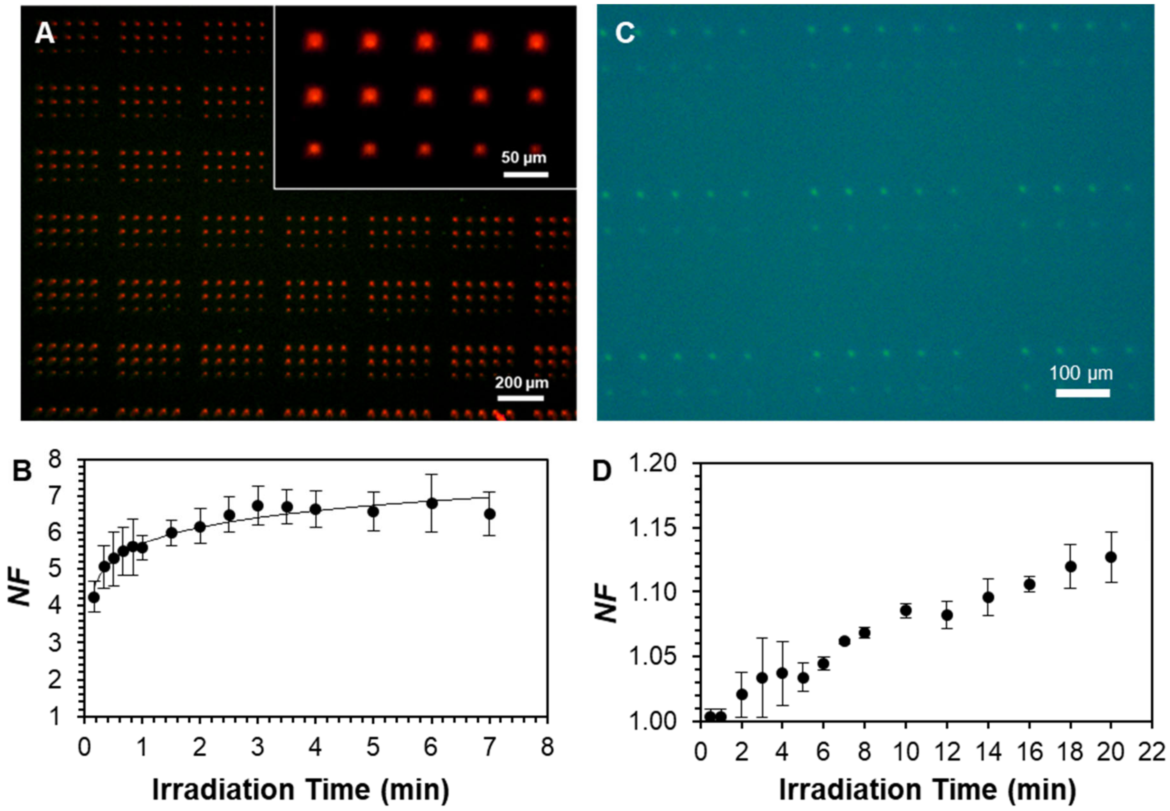

Supplementary Figure 6. RMA and CMA optimization. (**A**) Fluorescence microscopy image (λ_{ex} = 530 – 550 nm, barrier filter λ_{em} = 575 nm) of a polymer brush pattern composed of 15 features of *p*(MMA-RMA) in molar ratio (5000:1) at different irradiation times (15 s – 7 min). Inset shows a magnified array. (**B**) Average normalized fluorescence intensity (five arrays) logarithmically increases with irradiation time (R^2 = 0.98). Error bars correspond to standard deviation of five measurements. (**C**) Fluorescence microscopy image (λ_{ex} = 330 – 385 nm, barrier filter λ_{em} = 438 nm) of a 15-feature pattern of *p*(MMA-CMA) in molar ratio (223:1) at 30 s to 20 min irradiation time. The analysis (**D**) reveals NF_{max} = 1.13 ± 0.02. Error bars correspond to standard deviation of five measurements.

6. Multicolor printing

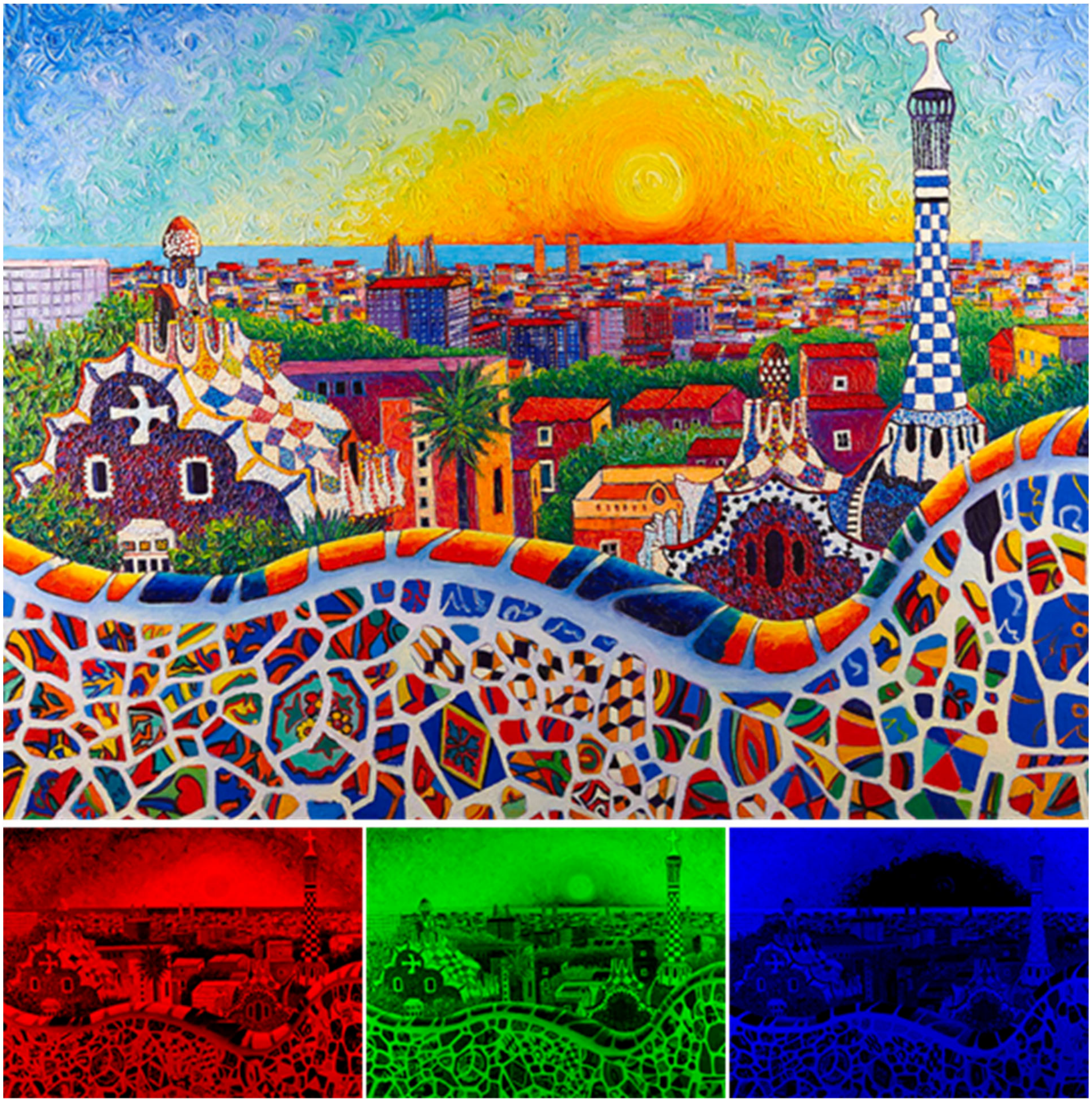

Supplementary Figure 7. Image template for Figure 3. Painting of Barcelona (This image is not covered by the article CC BY license**.** Image credit to Ms. Ana Maria Edulescu. All rights reserved, used with permission.) decomposed into Red, Green, and Blue (RGB) channels (below). Each channel will be used to generate polymer brushes of RMA, FMA, and CMA doped MMA, respectively.

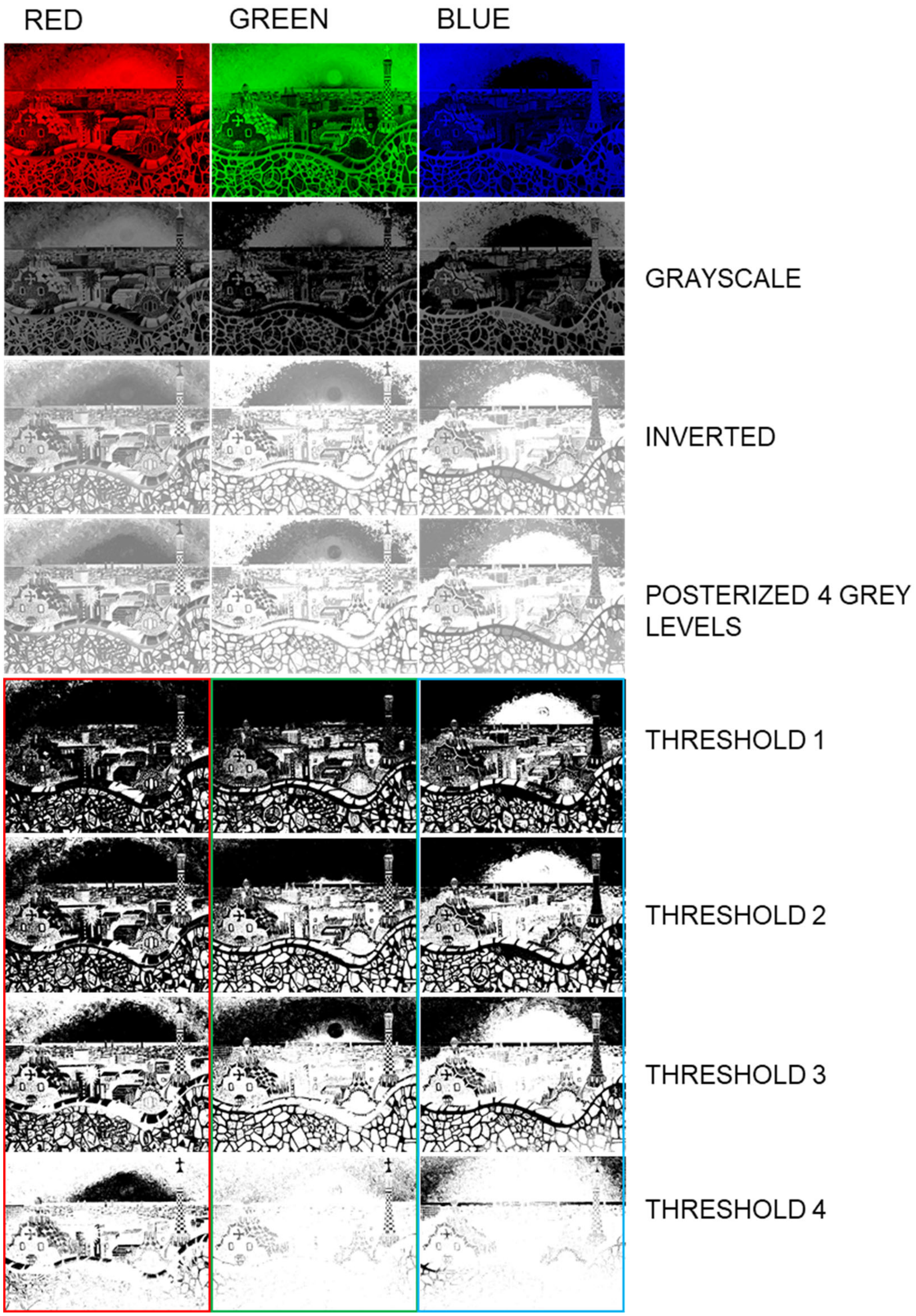

Supplementary Figure 8. Image preparation for printing Figure 3. Process to obtain the four black and white images used to indicate which mirrors project light at each time. Each one of the RGB channels is first transformed to a greyscale image, then, they are inverted and transformed to four grey levels. Finally, a threshold is stablished at each one of the four grey levels, generating four images that will be sequentially uploaded to the computer and to irradiate

during the four determined times. This image is not covered by the article CC BY license. Image credit to Ms. Ana Maria Edulescu. All rights reserved, used with permission.

7. Supplementary Methods

MALDI-IMS Analysis

MALDI-IMS Sample Preparation:

Unbound polymer was synthesized in the printer by irradiation of a solution of methyl methacrylate with photoinitiator in DMF for 1, 2, 5, 10, 20, and 40 min. Samples were dried and resuspended at 20 mg/mL in DMF for analysis via MALDI-IMS. ITO (indium-tin oxide) coated glass slides with 70-100 ohms resistivity (Bruker Daltonics) were used for all MALDI-IMS studies. Unbound polymer in DMF (1.5 µL) was pipetted directed onto the surface of the slide and allowed to dry. Polymer-bound printed surfaces were glued to the glass slide and 10 pieces of tape measuring 0.55 mm were used to equalize the height difference of both types of samples.

MALDI-IMS Analysis:

Slides were mounted into an MTP Slide Adapter II and loaded into a Bruker Autoflex III TOF MALDI mass spectrometer for analysis using flexControl software (Bruker Daltonics 8237001). Samples were analyzed by MALDI-MS under linear positive mode (4-20 kDa) using a 355 nm smartbeam 2 laser with a 50 µm focus diameter and 100 Hz frequency, a constant laser power of 54%, and a sum of 125 shots per spectrum. Spectra were collected within the region of 4060-20,000 Da using an accelerating voltage of 20 kV, and detector gain of 792 V. Region of interest (ROI) mapping and image analysis was performed in flex Imaging software (Bruker Daltonics). Raw mass spectra within a giving region of interest (ROI) were averaged and the baseline subtracted. No spectral normalizations were performed. For each 50 µm diameter pixel, integrated total signal from the mass spectra within a mass range filter of 5000 (+/- 500) *m/z* was calculated. Visual 2D maps were generated from these pixels, and colorized according to 0-100% maximal signal on a logarithmic scale.

Multicolor image treatment.

To compose the image in **Figure 3b**, we first placed the 25 fluorescence microscopy images taken with the red filter (λ_{ex} = 530 – 550 nm, barrier filter λ_{em} = 575 nm) as background (red composed image) (**Supplementary Figure 9A**). Then, we overlaid the green composed image (**Supplementary Figure 9B**) with 60% transparency and finally, blue composed image (**Supplementary Figure 7C**) was treated as explained below.

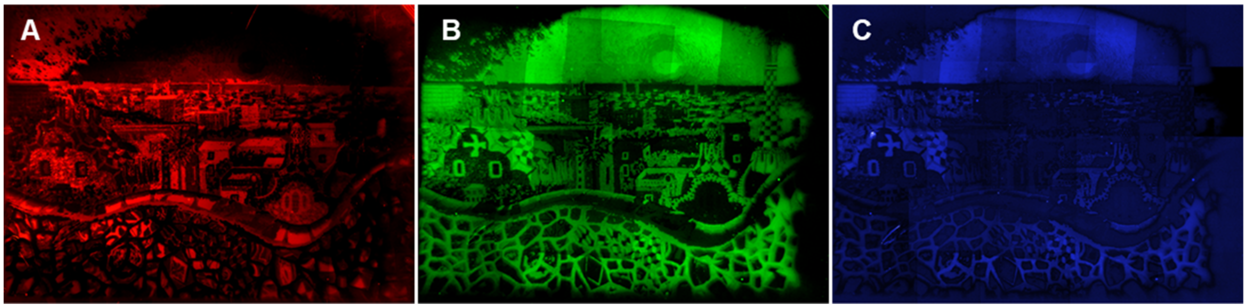

Supplementary Figure 9. **Fluorescent images used for Figure 3.** Fluorescence microscopy composition of 25 images obtained with filter sets for red (**A**), green (**B**) and blue (**C**).

The filter set for visualizing blue emitting fluorophore contains a long pass filter allowing the wavelengths >440 nm to pass through (**Supplementary Figure 10A**). RMA and FMA fluorophores both present certain absorption in the excitation range of the filter set (330 – 385 nm) and both also emit in the range of the mentioned long pass filter, so all three fluorophores are visualized when using the blue filter set (**Supplementary Figure 10B**). To show the emission generated by the CMA polymer, we distinguished the emission corresponding to FMA. As we can see from the b/w images used to indicate the DMD mirrors (**Supplementary Figure 10C-E)** there are almost no overlapping involving red. However, mirrors used to print blue and green polymers share most of the printed areas (**Supplementary Figure 10D-E**). We took the b/w image of the highest threshold used to print blue, and subtracted the b/w image of the highest threshold used to print green. Those b/w images correspond to the mirrors that have been on at any moment to print each specified color. Therefore, the result of the subtraction correspond to the mirrors that only have been used to print blue (**Supplementary Figure 10F**), and this image was used to show the blue regions of the blue composed image (**Supplementary Figure 10G**).

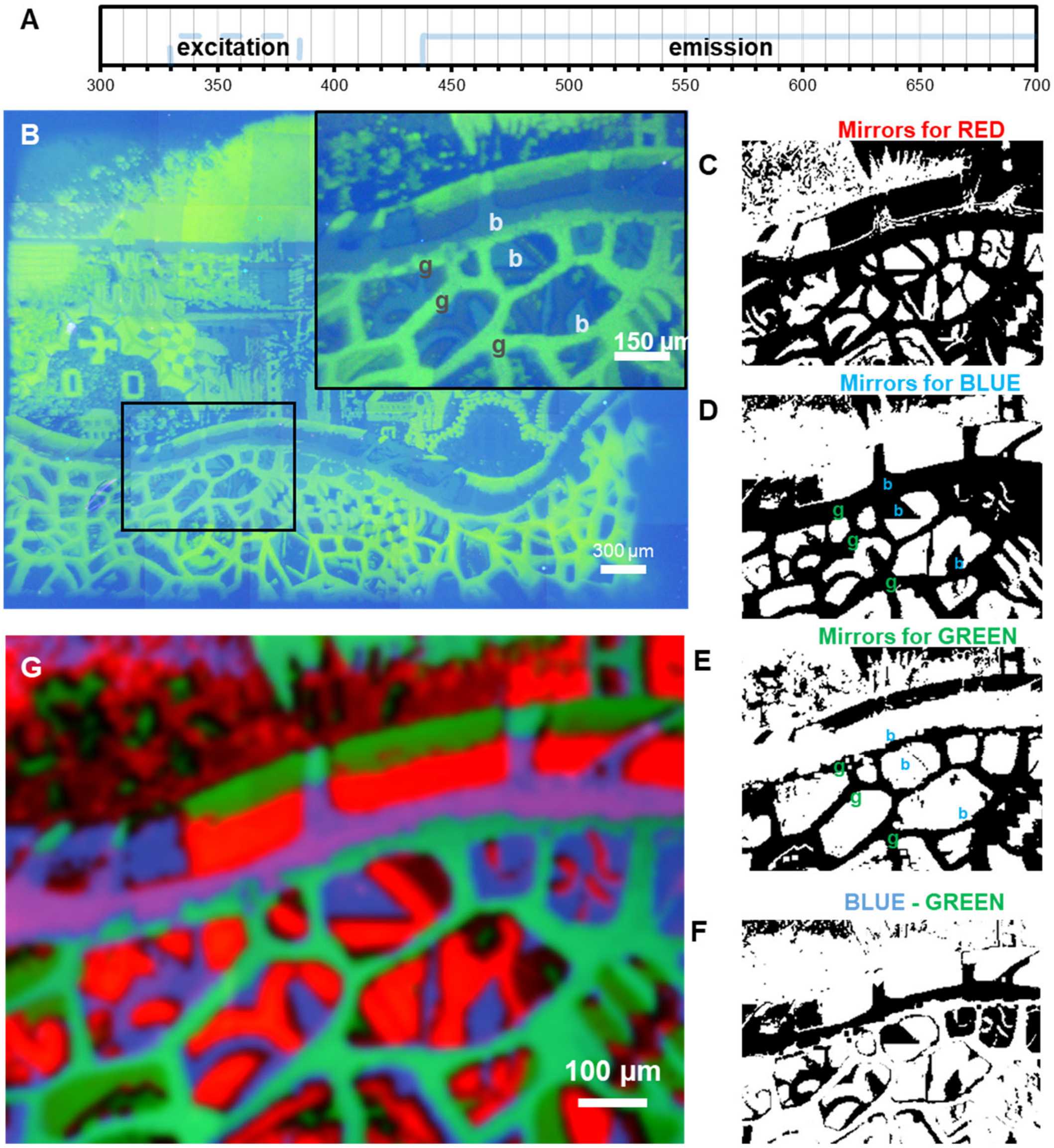

Supplementary Figure 10. **Fluorescence analysis for preparation of Figure 3.** (**A**) Blue emission filter set (**B**) Composition of 25 fluorescence microscopy images (λ_{ex} = 330 – 385 nm, and a barrier filter λ_{em} = 438 nm). Inset shows a magnified area where “g” indicates regions that contain green brushes and “b” regions that have been only irradiated when blue mixture was flowing. (**C**) Black pixels corresponding to DMD mirrors ON during red printing, blue printing (**D**) and green printing (**E**). Image used to turn mirrors ON during green and blue printing were subtracted (**F**) and used as a mask to show where only blue was printed (**G**).

8. Calculation of grafting density

Grafting density, σ (chains/nm^2), is determined from **Eqn. 1**

$$\boldsymbol{\sigma = \frac{H \cdot \delta \cdot N_A}{M_n}} \qquad \textbf{(Eqn. 1)}$$

where H is the polymer height in nm, N_A is Avogadro's number, M_n is the polymer number average molecular weight, and δ is the polymer density, with an accepted value of 1.1 g/mol for PMMA.[3]

Polymer height, H, was determined by two different methods:

PMMA Polymer brush height was determined by ellipsometry, while sacrificial initiator was placed in the solution above the initiator to determine M_n. This method of using sacrificial initiator in solution to estimate the M_n of brush polymers has been used widely to determine the molecular weights of grafted-from polymers.[4,5] Using this method, polymer brushes were grown from three different surfaces in three different experiments. To grow the polymers, a DMF solution with MMA, $Ir(ppy)_3$, and that had been doped with 30 μL (0.28 mmol) of the monomer ethyl α-bromoisobutyrate (EBIB) was placed onto a surface functionalized with the CTA. The surface was irradiated with the LED for 40 min, and heights of 14.9 ± 0.6, 15.0 ± 0.6, and 15.1 ± 0.6 nm were determined by ellipsometry using an IR-VASE ellipsometer and WVASE Spectroscopic Ellipsometry Data Acquisition and Analysis Sofware. The polymers from the supernatant that had been doped with 30 μL (0.28 mmol) of EBIB and irradiated for 40 min, and their M_n were determined by size exclusion chromatography multi angle light scattering (SEC-MALS) analysis to produce corresponding M_n (PDI) values of 7700 (1.15), 8100 (1.22), and 1300 (1.26) g/mol. Polymers were dissolved at 5 mg.mL^{-1} in DMF and analyzed by SEC-MALS with on a Phenomenex Phenogel 5u 103Å, 1K-75K, 300 x 7.8 mm in series with a Phenomenex Phenogel 5u 103Å, 10K-100K, 300 x 7.80 mm) at 65 ° C in 0.05 M LiBr in DMF, using a ChromTech Series 1500 pump equipped with a multi-angle light scattering detector (DAWN-HELIOS II, Wyatt Technology) and a refractive index detector (Wyatt Optilab T-rEX) normalized to a 30000 MW polystyrene standard at a flow rate of 0.75 mL/min.

Using Eqn. 1 resulted in σ values of 1.3 (1.1), 1.2 (1.0), and 7.9 (6.2) chains/nm^2 with numbers representing σ values calculated using M_n and (M_w). The third value was an outlier and physically impossible, so this number was discarded to produce an averaged value of 1.3 (1.1) chains/nm^2. This corresponds to a per-chain footprint, A_x, of 0.96 nm^2.

**Sample**	**$M_{n,\ SEC\text{-}MALD}$**	**$M_{W,\ SEC\text{-}MALS}$**	**PDI**
Sample I	7744	8975	1.159
Sample II	8088	9848	1.218
Sample III	1273	1608	1.264

To confirm that our ellipsometry experiments were determining film height accurately, we developed a new method to calculate brush heights. This process involved growing metal features of known height on a Si/SiO_2 wafer. After deposition of the metal features, the surfaces were functionalized with initiator and PMMA brushes were grown under the same conditions as

previously described. The premise of this experiment is that the polymers will only grow onto areas where there is no metal grown, and the height of the polymer films is determined by looking at the change in metal feature height before and after polymers are grown. This difference is the height of the brush polymers. Measurements on three independent substrates resulted in polymer film heights of 14.4 ± 6.3 nm, which is in good agreement with the height of the polymer films determined by ellipsometry (14.0 – 15.1 ± 0.6 nm).

9. References

1. Schneider, C. A., Rasband, W. S. & Eliceiri, K. W. NIH Image to ImageJ: 25 years of image analysis. *Nature Methods,* **2012** *9*, 671.

2. Lura, T.; Ohtani, H. Fragmentation behavior of poly(methyl methacrylate) during matrix-assisted laser desorption/ionization. *Rapid Communications Mass Spectrometry*, **2015** 155-62.

3. Shah, R. R.; Merreceyes, D.; Husemann, M.; Rees, I.; Abbott, N. L.; Hawker, C. J.; Hedrick, J. L. Using Atom Transfer Radical Polymerization To Amplify Monolayers of Initiators Patterned by Microcontact Printing into Polymer Brushes for Pattern Transfer. *Langmuir,* **2000**, *33*, 597-605.

4. Zoppe, J. O.; Ataman, N. C.; Mocny, P.; Wang, J.; Moraes, J.; Kolk, H.-A. Surface-Initiated Controlled Radical Polymerizations: State-of-the-Art, Opportunities, and Challenges in Surface and Interface Engineering with Polymer Brushes. *Chemical Reviews*, **2017**, *117*, 1105-1318.

5. Narupati, Bl; Page, Z. A.; Treat, N. J.; McGrath, A. J.; Pester, C. W.; Discekici, E. H.; Dolinski, N. D.; Meyers, G. F.; Read de Alaniz, J.; Hawker, C. J. Simultaneous Preparation of Multiple Polymer Brushes under Ambient Conditions using Microliter Volumes. *Angew. Chem. Inter. Ed.* **2018**, *57*, 13433-13438.
